# Electrochemotherapy: A Review of Current Status, Alternative IGP Approaches, and Future Perspectives

**DOI:** 10.1155/2019/2784516

**Published:** 2019-01-03

**Authors:** Nazila Esmaeili, Michael Friebe

**Affiliations:** INKA Intelligente Katheter, Otto-von-Guericke-Universität Magdeburg, Magdenurg, Germany

## Abstract

The efficiency of electroporation (EP) has made it a widely used therapeutic procedure to transfer cell killing substances effectively to the target site. A lot of researches are being done on EP-based cancer treatment techniques. Electrochemotherapy (ECT) is the first EP-based application in the field of drug administration. ECT is a local and nonthermal treatment of cancer that combines the use of a medical device with pharmaceutical agents to obtain local tumor control in solid cancers. It involves the application of eight, 100*µ*s, pulses at 1 or 5000 Hz frequency and specified electric field (V/cm) with a median duration of 25 minutes. The efficacy of chemotherapeutic drugs increases by applying short and intense electrical pulses. Several clinical studies proposed ECT as a safe and complementary curative or palliative treatment option (curative intent of 50% to 63% in the treatment of Basal Cell Carcinoma (BCC)) to treat a number of solid tumors and skin malignancies, which are not suitable for conventional treatments. It is used currently for treatment of cutaneous and subcutaneous lesions, without consideration of their histology. On the contrary, it is also becoming a practical method for treatment of internal, deep-seated tumors and tissues. A review of this method, needed instruments, alternative image-guided procedures (IGP) approaches, and future perspectives and recommendations are discussed in this paper.

## 1. Introduction

When a sufficiently large electric field is applied to a tissue for an adequate duration, transmembrane voltage is induced across the cell plasma membrane. As a result, the changes in the geometrical and material properties of the tissue cause local deficiencies in the cell membrane and make it permeable to agents that otherwise could not transfer into it. This phenomenon is usually known as electroporation (EP) [1, 2]. There are two types of EP: reversible and irreversible. The duration of the pulses and electric field intensity determine whether the structural changes in the cell membrane are reversible, allowing cells to survive or irreversible, leading to cell death because of the loss of homeostasis [3].

This phenomenon became a common technique for loading cells with materials that are either not possible or difficult to pass through the cells. This led to the growth of EP-based technology for biomedical applications and researches in the field of drug delivery and gene therapy, like gene electrotransfection, nonthermal irreversible electroporation, and electrochemotherapy (ECT) [4–6]. The effectiveness of these kinds of therapies depends on two factors: firstly, the electrical characteristics such as amplitude and duration of electric pulses, number of pulses and repetition, and the type of electrodes which are used and, secondly, cell and tissues characteristics, like shape, size, and cytoskeleton structure and membrane composition, that want to perform the EP on it [7, 8].

ECT is a local and nonthermal tumor ablation modality, which combines the administration of a poorly permeant cytotoxic agent with the local application of electric pulses that induce reversible EP, thus improving drug diffusion into the cells. Through this method, the efficacy of chemotherapeutic medications increases by the use of electrical pulses which provides good local tumor control [9–11].

The underlying chemical and physical processes related to the efficiency of large electric fields to cells in the tissue are still not sufficiently clarified. However, the predictions of theoretical models and the results of experimental studies show that the application of an appropriate electric field to the tumor leads to the temporary opening of aqueous pores on the cell membrane which allows the passing of drugs, genes, or molecular probes [12].

**Figure 1:**
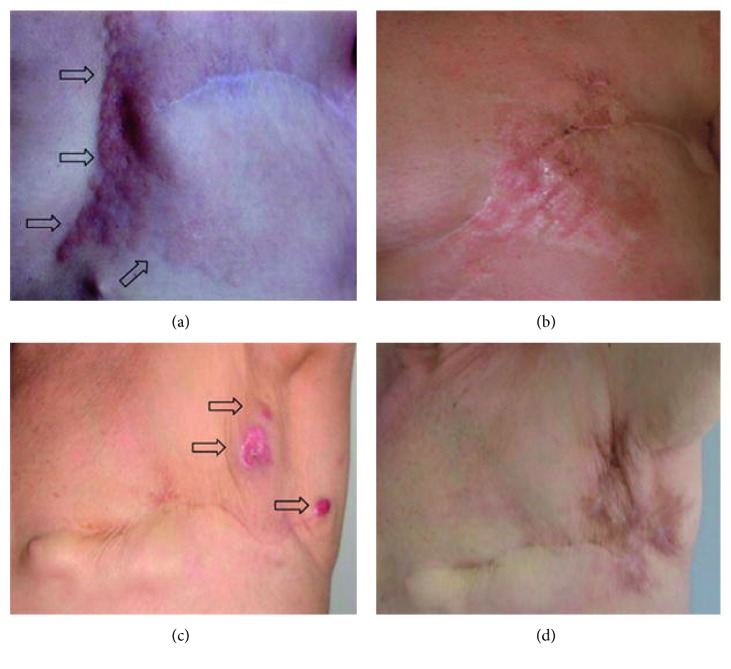
Skin metastases from breast cancer treated with ECT in two patients. (a, c) Before treatment. (b, d) 1-year follow-up. Arrows contour tumor spread or indicate skin metastases [10].

ECT effectiveness has been approved in different types of tumor. The first clinical study was published in 1991 on head and neck tumor nodules. ECT has been used in the treatment of subcutaneous and cutaneous lesions and metastases from tumors, with objective response ranging from 75% to 99%. It is applied for treating melanomas, sarcomas, and other types of skin cancer, cervix leiomyosarcoma, and breast cancer.  Figure 1 shows the result of an actual treatment using ECT [2, 13–15].

ECT can be used as an alternative approach or as a palliative treatment after standard therapies (such as surgery, radiotherapy, and chemotherapy) to improve the quality of life for patients. Recently National Institute for Health and Care Excellence (NICE) in UK suggests ECT to manage inaccessible or otherwise difficult to treat primary Basal Cell Carcinoma (BCC) and Squamous Cell Carcinoma (SCC) in carefully selected patients. It also supports ECT use as a palliative treatment for metastases in the skin from tumors of nonskin origin and melanoma, with normal arrangements for clinical governance, consent, and audit. Furthermore, Arbeitsgemeinschaft Dermatologische Onkologie (ADO) guideline in Germany indicates sufficient evidence of ECT efficiency as an alternative approach in the treatment of loco-regional metastases from malignant melanomas [16–18].

**Figure 2:**
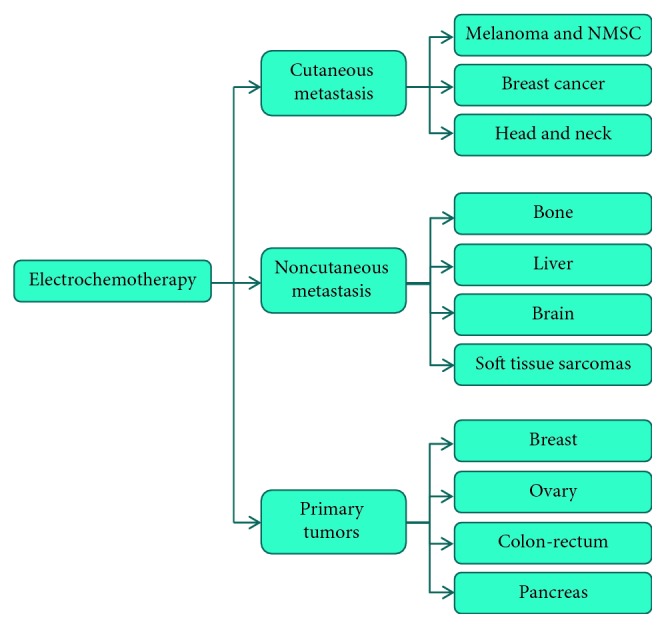
Current and potential application of ECT for tumor treatment.

Nowadays, ECT development is focused on treatment of mucosal, bigger, and deep-seated tumors and in internal organs or more centimeters below the skin.  Figure 2 shows the current and potential applications of ECT.

ECT has been known by some of national health services for the treatment of patients with cutaneous metastases from various tumor histotypes. It has been adapted over 150 cancer centers throughout Europe and has contributed to its diffusion in clinical practice. Also in Europe several dedicated patient's databases exist, in which International Network for Sharing Practice in ECT (INSPECT) database and Italian Melanoma Group and Gruppo Italiano Dermatologico Oncologico (GIDO) are two most important ones. INSPECT was established to investigate the effectiveness and utility of ECT as well as patient management by collecting prospective data and to share experiences and improving the clinical practice. The Italian Melanoma Intergroup (IMI) and GIDO prompted a prospective, multicenter, and observational study to evaluate the efficacy treatment of ECT. 376 eligible patients with superficial metastases, who underwent ECT from October 2008 through March 2013, were investigated. The results of this study strongly supports the efficiency of ECT in patients with superficial metastases from any histotype and skin cancer [11, 14, 19–23].

Following a review of the ECT procedure in the terms of practical implementation and technical requirements, the new application of ECT, alternative Image-Guided Procedures (IGP) approaches, and what needs to be done for further application are discussed.

## 2. Practical Implementation and Technical Requirements

### 2.1. Principles and Treatment Procedure

**Figure 3:**
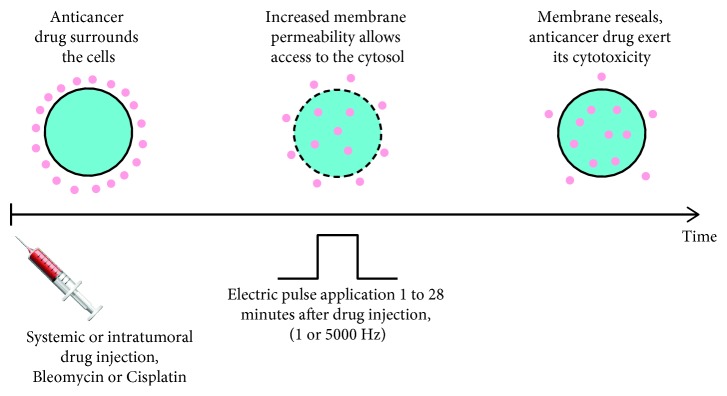
Principle of ECT.

ECT is a procedure that combines a membrane nonpermeant or poorly permeant cytotoxic drug having an intracellular target with the use of short and intense electric pulses which are causing increased membrane permeability [11, 24]. The basic principles for effective treatment are as follows. First, the pharmacological peak of the injected cytotoxic drug reaches the tumor at the time of application of short and intense electric pulses (the range between reversible and irreversible electropermeabilization threshold values). Second, the entire tumor mass is sufficiently covered by the electric field (Figure 3) [2, 25].

ECT is performed using either intratumoral or intravenous cytotoxic drug injection, followed by the application of electric pulses locally delivered to the target tumor via suitable sets of electrodes. When the external electric charges are applied to the cells, charges on the cell membrane start to move. This causes the gradual increase of transmembrane voltage (called induced transmembrane voltage, which is added to the cells' normal resting transmembrane voltage or resting membrane potential). An adequate and strong electric field is established through the cell membrane, where transmembrane voltage goes over the limit of a particular voltage. This leads to the establishment of a passage (pore) for water, charged and larger molecules to go across the cell membrane. They are created rapidly and disappear within a few seconds to several minutes after exposure to the electric field. Although these pores are too small and short-lived to be observed using conventional or electron microscopy, indirect evidence supporting their existence comes from simulations of lipid bilayers using molecular dynamics simulations. The mechanism of membrane resealing or repair requires active cellular mechanisms and therefore also energy. So, a possible explanation of the relatively long-term permeability of cell membranes is the chemical alteration of membrane lipids [7, 26].

Studies showed that the main focus of the chemotherapeutic agents is on the actively dividing cancer cells and less on the nondividing population of normal cells in the surrounding tissue. When the tumor tissue is exposed to the electrical pulses, the tumor blood flow will reduce up to 80% and will come back to normal flow within 24 hours. In this way, the injected drug can stay in the tumor tissue for several hours which gives it more time to act [2, 4].

Although ECT efficacy both in human and veterinary oncology was well demonstrated, differences among treatment protocols, the lack of defined operating procedures, and the use of different pulse generators prevented the widespread adoption of ECT in the clinical setting for a while [5, 26]. There was a critical need to have extremely accurate procedures for either systemic or local cytotoxic drug delivery, followed by the application of electric pulses, and also for each specific clinical condition [2].

The first version of standard operating procedure (SOP) has been developed and validated by the European Standard Operating Procedures for ECT (ESOPE) in 2006. This reference describes the recommendations for indication of ECT, pretreatment information, treatment choices, and follow-ups in order to safely and conveniently treat patients with cutaneous and subcutaneous nodules. The SOP decision tree is to help the clinical staff to make decisions on how to treat the patient, based on the size, number, and thickness/depth of nodules to be treated. The evaluation and confirmation of treatment response based on ESOPE showed 85% objective response rate and 74% complete response rate of ECT, regardless of tumor histology, drug used, and route of drug administration. Recently, a new version of SOP for ECT of cutaneous tumors and skin metastasis is published by a Pan-European expert panel from dermatology, general surgery, head and neck surgery, plastic surgery, and oncology. ECT procedure can be performed in 30 minutes both in local or general anesthesia, which should be decided by the treating surgeon, but few and smaller nodules are recommended to be treated in local, while others in general anesthesia [26–29].

### 2.2. Instruments

ECT is performed with the help of an electrical pulse generator and different types of electrodes. These instruments are explained in detail as follows.

### 2.3. Electric Pulse Generator

EP of cells is predominantly induced by pulsed electric fields, through a train of square wave electric pulses of sufficient amplitude establishing a local electric field (hundreds of V/cm) [3], and applied to the tumor cells with the help of electrodes, in order to let EP phenomenon happen [27].

Adequate pulse amplitudes were initially and are all too often still determined empirically. In clinical settings, monopolar, direct current, and short and intense square-shaped electric pulses are used. In order to predict electric field distribution in biological tissues, numerical modeling is currently the only efficient way, as these are characterized by inherently nonlinear, nonhomogeneous, and, in some cases, anisotropic dielectric properties. Based on initial experiments in the laboratory, preclinical, and clinical studies for the treatment of cutaneous accessible tumor nodules, 8 pulses of 100 *µ*s duration delivered at 1 Hz or 5000 Hz repetition frequency. The voltages applied depend essentially on the electrode type, number of electrodes, and electrode geometries like distance between the electrodes and are automatically set by the pulse generator. For the treatment of tumor nodules accessible by fibroscopy, endoscopy, laparoscopy, or open surgery, SOP and equipment are still under development [7, 27, 30, 31].

### 2.4. Types of Electrode

**Figure 4:**
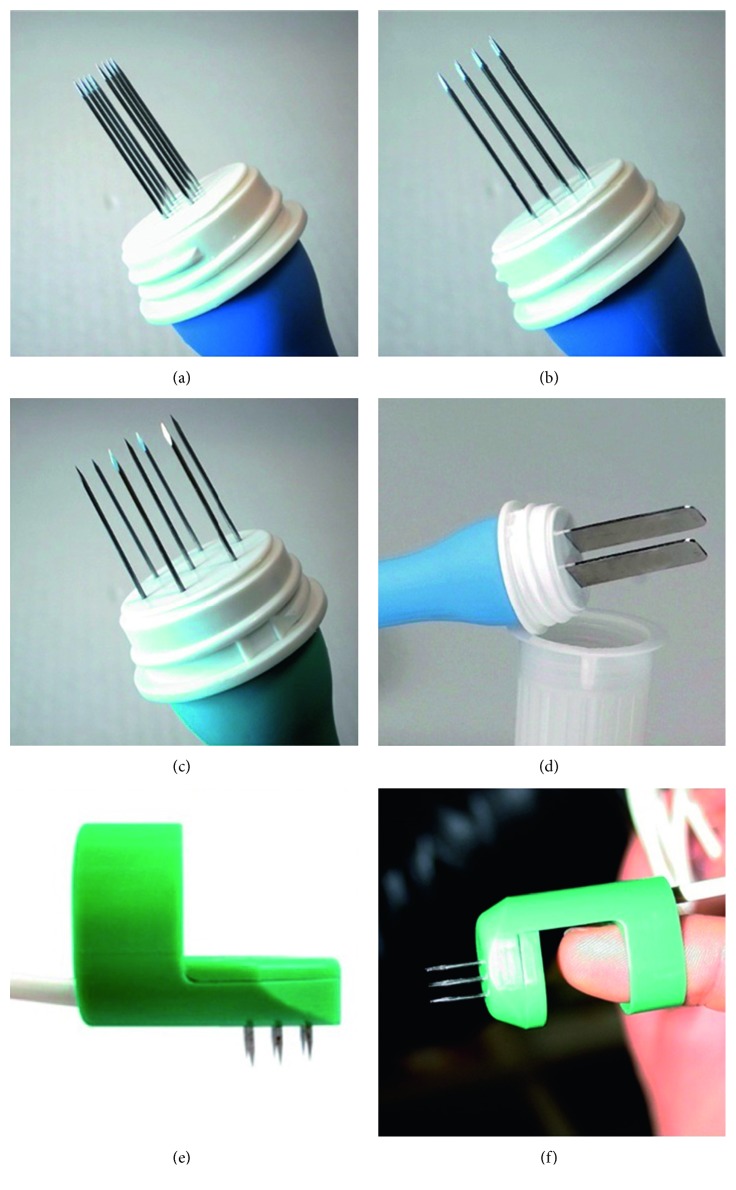
(a) Linear needle electrodes, 10 to 30 mm length, recommended voltage 400 V. (b) Hexagonal needle electrodes, 10 to 40 mm length, recommended voltage 730 V. (c) Plate electrodes, 30 mm length, recommended voltage 960 V. (d) Finger electrodes with perpendicular needles, 5 to 10 mm length recommended voltage 400 V. (e) Finger electrodes with axial needles, 5 to 10 mm length recommended voltage 400 V [26, 32].

Different sets of electrodes for different nodules with different depths, sizes, and shapes are available, either plate or needle electrodes. Different electrode configurations are used to enable sufficient electric field coverage of the whole tumor volume.  Figure 4 illustrates different types of electrodes [11].

Plate electrodes are applied for the treatment of superficial or skin lesions. The distance between the electrodes affects the penetration depth of the electric field. A large voltage needs to be administered between plate electrodes with great distance to penetrate the electric field deep into the tissue. Needle electrodes are of two kinds: needles are positioned either in parallel rows with a 4 mm gap between them, suggested for treatment of small nodules, or in a circular (hexagonal) array suggested for bigger (>1 cm in diameter) nodules. In contrast to plate electrodes, needle electrodes can be placed through the tumor tissue, up to 3 cm deep [2, 7, 27].

**Figure 5:**
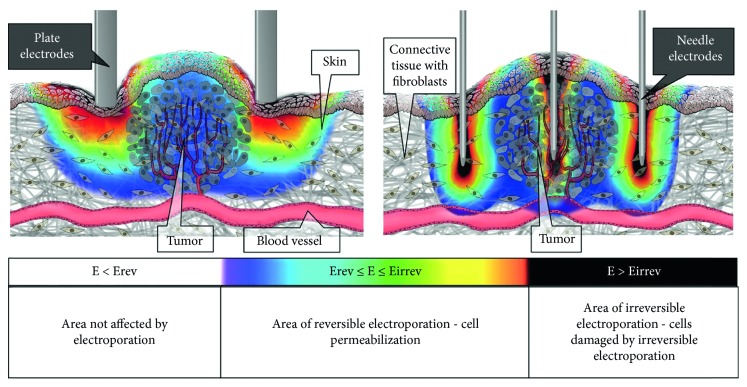
Electric field distribution in and around the tumor tissue during the application of electric pulses for plate (left) and hexagonal needle electrodes (right). The distribution is indicated with the rainbow color scale [19].

One important point is that aside from the type of electrode, the electric field is highest around the electrode and between the electrodes but lowers rapidly outside the electrode array (Figure 5). Therefore, the whole tumor can only be treated in an efficient manner by moving and placing electrodes nearby for each sequential electric pulse application, if the tumor is larger than the distance between the electrodes. Because of the structural heterogeneity of tissues, the electric field that should be set in tumors for the permeabilization of cells is hard to determine. Thus, it is important to use the suggested pulse amplitude for different types of electrodes. This information is provided by the manufacturers of clinical electroporators [2, 19].

### 2.5. One Example

**Figure 6:**
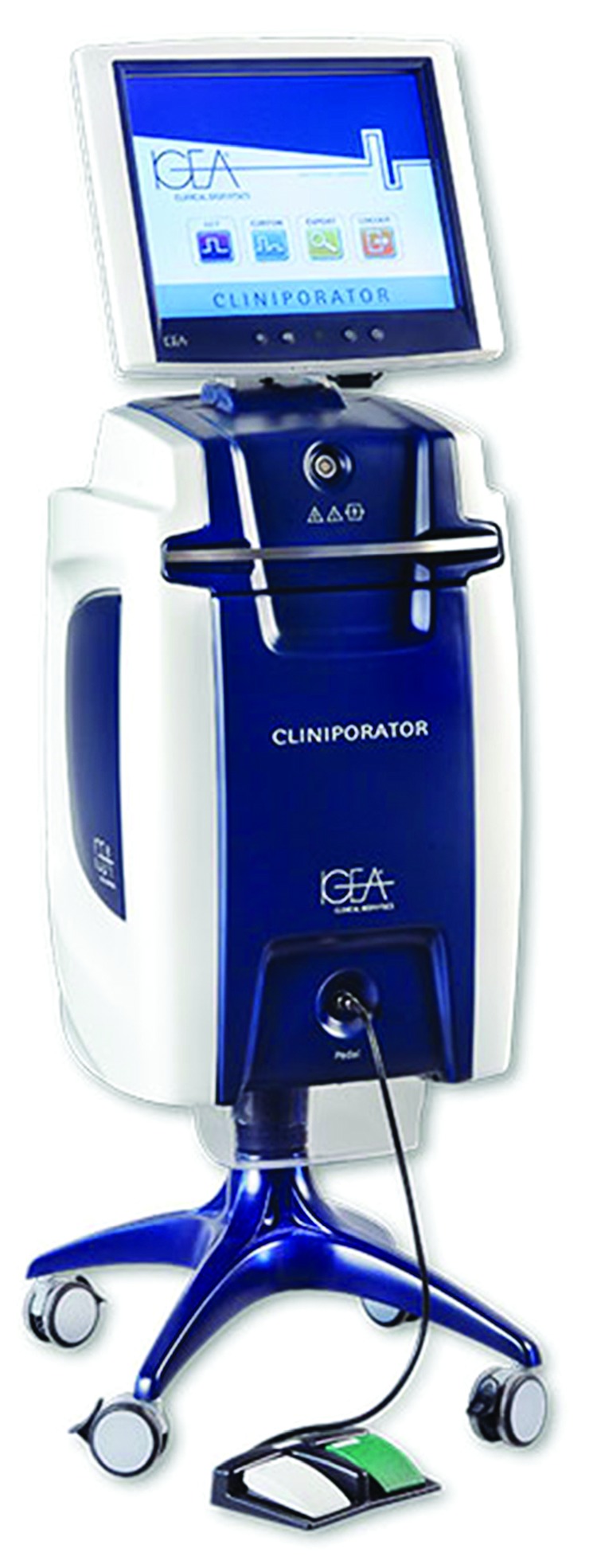
The electric pulse delivery unit Cliniporator™ [32].

The Cliniporator™ (IGEA, Carpi, Italy) was introduced by ESOPE to perform ECT in clinical practice. This device is used in several countries around the world. Cliniporator™ consists of a console unit, a power supply unit for supplying the current, and an applicator (electrodes) for placement on the skin. This device is computer controlled. Additionally, this is the only device that offers the control of the pulses delivered on a screen, just after the delivery of the pulses. Therefore, the effectiveness of each individual EP can be observed on the monitor to prevent excess current for patient safety. The scheme of this device is illustrated in Figure 6 [33].

### 2.6. Cytotoxic Agents

Hydrophilic and nonpermeant or poorly permeant chemotherapeutic drugs are suitable for ECT. Hence, several drugs were tested on cells to find the best combination with EP; these include daunorubicin, doxorubicin, etoposide, paclitaxel, etoposide, actinomycin D, Adriamycin, mitomycin C, 5-fluorouracil, vinblastine, vincristine, gemcitabine, cyclophosphamide, carboplatin, cisplatin, and bleomycin. However, between all candidates only two of these drugs have been identified to date for ECT of cancer patients: bleomycin and cisplatin [2, 33].

Bleomycin can be delivered intravenously (15,000 IU/m^2^ in a bolus over 30–60 s) or locally through intratumoral injection. Intratumoral bleomycin should be administered in the form of the solution (1000 IU/ml), in a dose of 1000 IU/cm^3^ in lesions < 0.5 cm^3^, 500 IU/cm^3^ in lesions ≥ 0.5 cm^3^ and ≤1 cm^3^, and 250 IU/cm^3^ in lesions > 1 cm^3^. Cisplatin (2 mg/ml) is only administered intratumorally. The dose of cisplatin also depends on the size of neoplastic lesions. Lesions larger than 1 cm^3^ should be treated with a dose of 0.5 mg/cm^3^, those ≥0.5 cm^3^ and ≤1 cm^3^ should be treated with a dose of 1 mg/cm^3^, and for tumors smaller than 0.5 cm^3^, a dose of 2 mg/cm^3^ should be applied [5].

When the drug is administered systemically, electric pulses should be delivered to the tumor site during the pharmacokinetic peak, which was reported to be between 8 and 28 minutes in humans. For intratumoral application, however, the pulses need to be delivered from 1 min to 10 min after drug injection [2, 11, 27].

## 3. Advantage and Disadvantage of ECT

### 3.1. Advantages

ECT can be used when surgery is not an option. Also, it can be used for chemotherapy-resistant and radiotherapy-resistant lesions. ECT has comparable or superior effectiveness over several ablative skin-directed therapies such as over photodynamic therapy, radiotherapy, intralesional therapy, and topical therapy. ECT is suitable for patients with severe comorbidity and/or patients of an advanced age who have already exhausted all other treatments. Hence, it can improve the quality of patients' life by managing and controlling of various malignancies. The procedure is easy and quick to perform (25–30 minutes) and is associated with a short hospital stay. The side effects are minor and most patients do not require analgesics. In contrast to radiotherapy, it is possible to repeat several ECT cycles without precluding other types of treatment on the same patient if new metastases develop. ECT has a favorable cost-effectiveness ratio with a cost of € 1,901.05 per achieved response in comparison with other techniques to control and treat cutaneous and subcutaneous melanomas [13, 19, 24, 33, 34].

### 3.2. Disadvantages

In the case of using general anesthesia, the procedure is associated with common anesthesia-related risks. The use of intravenous bleomycin can cause pulmonary fibrosis, particularly when this treatment is administered to patients who have previously received radiation therapy. Due to increased tumor decomposition in the case of extensive ECT in particular, the formation of large ulcers is possible. It is possible that the ECT will have to be repeated. For instance, second ECT cycle in patients with small BCC can overcome some issues like recurrent representation, locally advanced diseases and aggressive histology to achieve complete response. Hence, consideration of whether an individual's life expectancy is long enough to obtain benefit from therapy and whether treatment will be adequately tolerated is required. Also in earlier studies, ECT was not recommended in patients with cardiac pacemakers and patients on anticoagulant therapy for safety reasons [2, 24, 33, 35].

## 4. New Applications of ECT

New studies tried to add new characteristics to ECT, in order to make it more effective for treatment of different types of cancer. Here, a short review of these studies is provided:

### 4.1. Large Tumors

In 2014, the prototype of a new grid electrode was proposed. This electrode is designed for treating large, tumor-infiltrated skin surfaces and is tested on the breast cancer patients with chest wall metastases after mastectomy. According to the tests in various in vitro models, this new electrode provides more quick and homogeneous voltage pulses in comparison with standard pulse applicators. Also, it can be easily connected to an existing and widely adopted electric pulse generator. Furthermore, this new grid electrode showed an improvement in ECT application [12].

### 4.2. Deep-Seated Tumors

Recently some researchers have focused on treating nonsuperficial tumors using ECT, like liver and bone metastasis, to treat them with the help of minimally invasive procedures. The main point is that, in these situations, procedures become more complex. In a comprehensive review of advanced techniques for deep-seated tumors' treatment based on EP in 2015, the current challenges in performing these treatments for these types of tumors were named as follows:

1. Determination of tissue conductivity (necrotic regions, vascular system, and microheterogeneities)
2. Amount and dynamics changes of conductivity due to EP
3. Determination of threshold for reversible and irreversible EP, also as a function of duration and number of pulses
4. The accuracy for treatment planning and positioning of electrodes

Hence, an overall evaluation of imaging, treatment planning, and clinical feedback is needed to make ECT available for clinicians around the world and deliver the best outcome and benefit to the patients [1].

In another study in 2014, clinical experiments and perspective of the use of ECT were investigated. The following aspects were mentioned as important points for nonsuperficial metastasis:

1. A robust software is needed for the treatment planning that modifies the electrode positions and electric pulse characteristics based on each patient
2. An accurate positioning of electrodes, such as long needles, require highly skilled surgeons as well as intraoperative images such as X-ray, ultrasound, or CT (computed tomography) imaging
3. The time interval between examination and image interpretation need to be defined for monitoring of ECT effect using CT, MRI (magnetic resonance imaging), or CT/PET (positron emission tomography).
4. The treatment of metastases localized in the abdomen requires synchronizing the pulse delivered to the heart's absolute refractory period to avoid interference with the heart's electrical activity [7]

**Figure 7:**
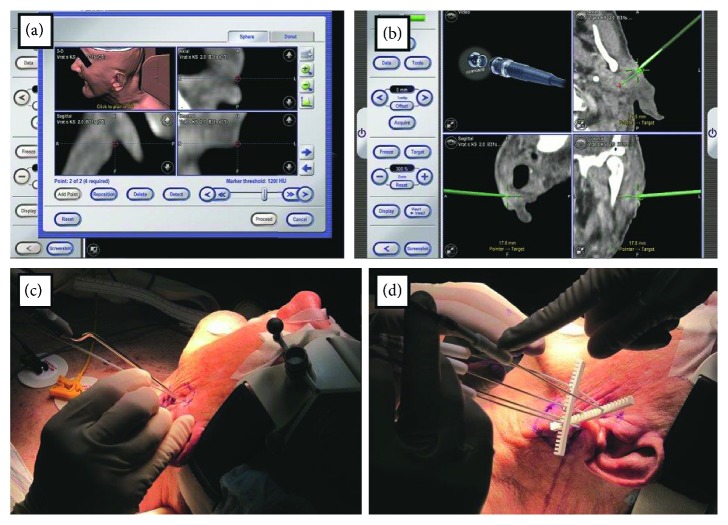
(a): Treatment 3-D planning for registration and navigation. (b) An Optical navigation was used to accurately access the planned skin entry points and direction of electrodes. (c) Positioning of navigation system needle and first needle electrode. (d) The final positioning of all five needle electrodes [5].

In order to help localizing the electrode precisely, a new technological approach in 2015 was introduced in the treatment of deep-seated head and neck tumors by ECT with the help of a treatment planning and navigation system. Long single needle electrodes were used to perform the procedure and also a treatment plan that provided the information on the optimal configuration of the electrodes to adequately cover the tumor with the electric field (Figure 7). Based on the results, the navigation system can provide accurate information for surgeons to identify the precise location of the tumors and can help them to position the long needle electrodes according to the treatment plan [5].

The effect of ECT of deep-seated tumors located close to the heart on the functionality of the heart was investigated in 2015 for the first time. During this study, changes in several electrocardiographic signals and heart rate variability parameters of patients with colorectal liver metastases treated with ECT were evaluated. No major heart rhythm changes (i.e., induction of extrasystoles, ventricular tachycardia, or fibrillation) or pathological morphological changes (i.e., ST-segment changes) indicating myocardial ischemia could be observed. Statistically significant but clinically irrelevant minor changes in heart rate and long-term heart rate variability parameters could be detected during intra-abdominal ECT, revealing that there are no life-threatening effects for the patients [6].

### 4.3. Pulsed Electromagnetic Field

For the first time in 2016, ECT was performed using pulsed electromagnetic field (PEMF) to treat mouse melanoma in vivo. For this, noninvasive EP was performed using a magnetic field pulse generator connected to an applicator consisting of a round coil. Subcutaneous mouse B16F10 melanoma tumors were treated with intravenous injection of cisplatin (4 mg/kg), PEMF (480 bipolar pulses, at the frequency of 80 Hz and pulse duration of 340 *μ*s) or with the combination of both therapies (ECT–PEMF + cisplatin). At the end, the results in mouse melanoma model in vivo demonstrated the possible use of PEMF-induced EP for biomedical applications, such as ECT. The main advantages of EP mediated by PEMF are contactless and painless application, as well as effective EP compared to the previously described conventional one [3].

## 5. Alternative Image-Guided Procedure (IGP) Approaches

ECT is one of the available techniques for local treatment of internal tumors. Other local therapeutic options include surgery and radiotherapy, which are prevalent, and thermal ablation techniques. Radiofrequency ablation (RFA) and microwave ablation (MWA) are two examples of thermal ablation techniques which can be performed minimally invasively. Furthermore, cryosurgery and laser therapy can be other options for local cancer treatment [1, 17, 24]. A comparison between ECT, RFA, and MWA is provided as follows:

1. These three techniques work with completely different principles. ECT works based on nonthermal reversible EP and combines the low-permeable cytotoxic drug with the application of electric pulses to kill the tumors [24]. RFA and MWA both are thermal tumor ablation techniques, but their heating mechanisms are quite different. In RFA, a needle electrode delivers an electrical current in the radiofrequency range under imaging (ultrasound, CT, and MRI) or surgical guidance. This produces heat-based thermal cytotoxicity based on a creation of complete electrical circuit through the body. The critical tissue properties for RFA are electrical conductivity and thermal conductivity. On the other side, MWA is a special case of dielectric heating, where the tissue acts as the dielectric material. Dielectric heating occurs by the application of an alternating electromagnetic (EM) field to an imperfect dielectric material. The EM field causes the oscillation of the water molecules which creates the heat in the tissue. Hence, the best heating effect is achieved in solid organs with a high content of water and the worst heating effect is observed in fat. Another mechanism of the MWA function is the conversion of kinetic energy into heat based on the concept of ionic polarization. In this manner, a larger and more homogeneous ablation zone that is easily predicted is achievable and the heat sink effect is decreased. The important properties of MWA are relative permittivity and effective conductivity. It is important to denote that effective conductivity in MWA and electrical conductivity in RFA are different concepts. Radiofrequency electrical conductivity relates to an alternating flow of electrons, while effective conductivity includes effects associated with the rotation of dipoles. Hence, having an electrically conductive path id is necessary for RF heating. Thus, microwaves can propagate through materials with low or zero conductivity [36, 37].

(2) The main focus of ECT is to control and treat different cutaneous and subcutaneous lesions and metastasis. Only in the past few years, it became a practical method for treating internal and deep-seated tumors [1]. RFA is mainly used to treat tumors in internal organs like liver, lung, kidney, and bone. Then, because of some limitation of RFA which affects its efficiency, MWA becomes a new technique for this kind of tumors [36].
(3) In the case of temperature, the temperature in RFA is between 60°C and 100°C and results in almost instant coagulation necrosis (low intratumoral temperature). For the MWA, the temperature can exceed 150°C (high intratumoral temperature) [36, 37]. However, due to the heat sink effect, thermal ablation techniques are not recommended in all situations. For instance, RFA is not recommended in the vicinity of major hepatic vessels and leads to frequent tumor recurrences [4]. In the thermal ablation of lungs, considerable blood flow and the nature of the lungs parenchyma are the causes of heat sink effect in lungs because they dissipate the applied heat and temperature change during thermal ablation treatment [38]. Therefore, ECT provides a nonthermal approach for the treatment of tumors in such locations. This is one of the main advantages of ECT in comparison with thermal ablative methods.
(4) The frequency range of ECT is lower than of the other ablative modalities (1 or 5000 Hz). RFA applies medium frequency alternating current (350–500 kHz), and MWA uses frequency between 900 and 2450 MHz [27, 36].
(5) Another point is that between these three modalities, MWA is the only technique that can treat multiple lesions simultaneously [37].
(6) Another major advantage of ECT over RFA and MWA is that ECT is nondestructive to the underlying tissues. Thermal ablative methods cause tissue destruction and necrosis to both tumor and normal tissue in the area of treatment. However, the applied electric pulses in ECT only act as a vector to increase or improve the internalization of the drug, which cause no damage to the underlying tissue. For example, a large number of patients with lung cancer are smokers who have a limited pulmonary function. Hence, any damage to the practical part of their lungs can result in sever pulmonary compromise. Moreover, preclinical studies of bone cancer treatment showed that, in ECT bone, osteogenic activity and structural integrity are preserved differently from thermal ablation techniques [38, 39].
(7) The effectiveness and safety have brought ECT into guidelines for the treatment of different cutaneous and subcutaneous tumor. In the case of internal and deep-seated tumors and from the point of view of the clinicians, the main challenge of applying ECT is that currently this procedure takes too long time in comparison with other minimally invasive procedures such as RFA or MWA. This is because of the requirement of multiple needles placement for ECT, while in RFA and MWA, only one probe is used [1].

## 6. Make ECT Applicable for Other Applications in the Future

ECT is now at the stage of palliative and curative treatment in most indications, or in the case other treatments have failed. It is in its early phase of clinical acceptance, and currently, the focus is on skin tumors and metastasis. However, further work is required. In this regard, treating deep-seated tumors needs more effort, time and resources. First, the effectiveness of applying ECT in comparison with other treatments should be considered. Because as it was mentioned before, placement of ECT needles is more complicated and time-consuming than other IGP approaches. On the other side, even though the technology of image-guided transcutaneous insertion of electrodes is available, pretreatment planning development for ECT is still in its early development. Hence, designing new electrodes and needles, using imaging modalities and treatment planning, can be helpful to develop ECT in the clinical practices. Besides of adding new features to the conventional form of this approach, the usability and price should be considered.

One idea to develop the use of ECT and also have a successful tumor treatment is to combine this technique with other modalities like surgery. Using ECT before the surgery to downsize the tumor can facilitate surgical interventions. Additionally, ECT would be used after surgical removal of the tumor to treat remaining mass. Furthermore, based on the several reports from animal models, another combination can be the use of ECT as radiosensitizing approach. Therefore, ECT could, by increased intratumoral accumulation, potentiate radiation response of tumors without normal tissue damage [26]. One of the novel ideas is also to combine ECT with other EP-based methods like immunomodulatory effect (immunogene electrotransfer) to increase the systemic antitumor effectiveness of ECT, which is on a great investigation to make it applicable for the clinical practices [19].

ECT is one EP-based method just for cancer control. As another idea, one possibility in the future might be the use of EP-based therapies in order to deliver the medications directly to the special organs or parts of the body, which are fighting with special diseases apart from cancer. Therefore, changing the components and chemical structures of the drugs in the case of the disease with limited treatment options will not be a problem in the future because we know how to administrate these drugs. This idea needs a comprehensive study on requirements to modify the instruments, especially for having a minimally invasive procedure.

## 7. Conclusion

Besides some limitations of ECT, studies showed that it is an effective treatment of tumor lesions located in the skin or subcutaneous tissue, both primary and metastatic, regardless of the histological type of the tumor or previous treatments. It has also shown its effectiveness in the case of treating deep-seated tumors. On the contrary, ECT offers considerable advantages, like in terms of time of hospitalization; it can be performed at a day hospital. It is also possible to repeat several ECT cycles without precluding other types of treatment. It has good cost/benefit ratio, and no serious adverse events or severe toxicity has been related to this technique.

However, because of the novelty of this method, more research and investigation are needed to develop ECT and make it more applicable for different types of cancers, as well as other diseases.

## References

[1] Miklavcic D., Rafael V. D. (2015). Electrochemotherapy (ECT) and irreversible electroporation (IRE)-advanced techniques for treating deep-seated tumors based on electroporation. *BioMedical Engineering OnLine*.

[2] Sersa G., Miklavcic D., Cemazar M., Rudolf Z., Pucihar G., Snoj M. (2008). Electrochemotherapy in treatment of tumours. *European Journal of Surgical Oncology (EJSO)*.

[3] Kranjc S., Kranjc M., Scancar J., Jelenc J., Sersa G., Miklavcic D. (2016). Electrochemotherapy by pulsed electromagnetic field treatment (PEMF) in mouse melanoma B16F10 in vivo. *Radiology and Oncology*.

[4] Miklavčič D., Serša G., Brecelj E. (2012). Electrochemotherapy: technological advancements for efficient electroporation-based treatment of internal tumors. *Medical and Biological Engineering and Computing*.

[5] Groselj A., Kos B., Cemazar M. (2015). Coupling treatment planning with navigation system: a new technological approach in treatment of head and neck tumors by electrochemotherapy. *BioMedical Engineering OnLine*.

[6] Mali B., Gorjup V., Edhemovic I. (2015). Electrochemotherapy of colorectal liver metastases - an observational study of its effects on the electrocardiogram. *BioMedical Engineering OnLine*.

[7] Cadossi R., Ronchetti M., Cadossi M. (2014). Locally enhanced chemotherapy by electroporation: clinical experiences and perspective of use of electrochemotherapy. *Future Oncology*.

[8] Corovic S., Bester J., Miklavcic D. (2009). An e-learning application on electrochemotherapy. *BioMedical Engineering OnLine*.

[9] Edhemovic I., Gadzijev E. M., Brecelj E. (2016). Electrochemotherapy: a new technological approach in treatment of metastases in the liver. *Technology in Cancer Research & Treatment*.

[10] Cabula C., Campana L. G., Grilz G. (2015). Electrochemotherapy in the treatment of cutaneous metastases from breast cancer: a multicenter cohort analysis. *Annals of Surgical Oncology*.

[11] Sersa G., Cufer T., Paulin S. M., Cemazar M., Snoj M. (2012). Electrochemotherapy of chest wall breast cancer recurrence. *Cancer Treatment Reviews*.

[12] Castiello M., Dughiero F., Scandola F. (2014). A new grid electrode for electrochemotherapy treatment of large skin tumors. *IEEE Transactions on Dielectrics and Electrical Insulation*.

[13] Domanico R., Trapasso S., Santoro M., Pingitore D., Allegra E. (2015). Electrochemotherapy in combination with chemoradiotherapy in the treatment of oral carcinomas in advanced stages of disease: efficacy, safety, and clinical outcomes in a small number of selected cases. *Drug Design, Development and Therapy*.

[14] Kunte C., Letulé V., Gehl J. (2017). Electrochemotherapy in the treatment of metastatic malignant melanoma: a prospective cohort study by InspECT. *British Journal of Dermatology*.

[15] Mir L. M., Belehradek M., Domenge C. (1991). Electrochemotherapy, a new antitumor treatment: first clinical trial. *Comptes Rendus de l’Academie des Sciences. Serie III, Sciences de la vie*.

[16] National Institute for Health and Care Exellence (2014). *Electrochemotherapy for Primary Basal Cell Carcinoma and Primary Squamous Cell Carcinoma*.

[17] National Institute for Health and Care Exellence (2013). *Electrochemotherapy for Metastases in the Skin from Tumours of Non-Skin Origin and Melanoma*.

[18] Pflugfelder A., Kochs C., Blum A. (2013). Malignes Melanom S3-Leitlinie “Diagnostik, Therapie und Nachsorge des Melanoms”. *JDDG: Journal der Deutschen Dermatologischen Gesellschaft*.

[19] Sersa G., Teissie J., Cemazar M. (2015). Electrochemotherapy of tumors as in situ vaccination boosted by immunogene electrotransfer. *Cancer Immunology, Immunotherapy*.

[20] Coletti L., Battaglia V., De Simone P., Turturici L., Bartolozzi C., Franco F. (2017). Safety and feasibility of electrochemotherapy in patients with unresectable colorectal liver metastases: a pilot study. *International Journal of Surgery*.

[21] Rotunno R., Campana L. G., Quaglino P. (2017). Electrochemotherapy of unresectable cutaneous tumours with reduced dosages of intravenous bleomycin: analysis of 57 patients from the International Network for Sharing Practices of Electrochemotherapy registry. *Journal of the European Academy of Dermatology and Venereology*.

[22] Matthiessen L. W., Keshtgar M., Kunte C. (2016). Electrochemotherapy for breast cancer-results from the INSPECT database. *Annals of Oncology*.

[23] Campana L. G., Testori A., Curatolo P. (2016). Treatment efficacy with electrochemotherapy: a multi-institutional prospective observational study on 376 patients with superficial tumors. *European Journal of Surgical Oncology (EJSO)*.

[24] Benevento R., Santoriello A., Perna G., Canonico S. (2012). Electrochemotherapy of cutaneous metastasis from breast cancer in elderly patients: a preliminary report. *BMC surgery*.

[25] Corovic S., Zupanic A., Miklavcic D. (2008). Numerical modeling and optimization of electric field distribution in subcutaneous tumor treated with electrochemotherapy using needle electrodes. *IEEE Transactions on Plasma Science*.

[26] Miklavčič D., Mali B., Kos B., Heller R., Gregor S. (2014). Electrochemotherapy: from the drawing board into medical practice. *BioMedical Engineering OnLine*.

[27] Mir L. M., Gehl J., Sersa G. (2006). Standard operating procedures of the electrochemotherapy: instructions for the use of bleomycin or cisplatin administered either systemically or locally and electric pulses delivered by the Cliniporator™ by means of invasive or non-invasive electrodes. *European Journal of Cancer Supplements*.

[28] Marty M., Gregor S., Garbay J. R. (2006). Electrochemotherapy–an easy, highly effective and safe treatment of cutaneous and subcutaneous metastases: results of ESOPE (European Standard Operating Procedures of Electrochemotherapy) study. *European Journal of Cancer Supplements*.

[29] Gehl J., Sersa G., Matthiessen L. W. (2018). Updated standard operating procedures for electrochemotherapy of cutaneous tumours and skin metastases. *Acta Oncologica*.

[30] Miklavcic D., Corovic S., Pucihar G., Pavselj N. (2006). Importance of tumour coverage by sufficiently high local electric field for effective electrochemotherapy. *European Journal of Cancer Supplements*.

[31] Mir L. M. (2006). Bases and rationale of the electrochemotherapy. *European Journal of Cancer Supplements*.

[32] “Cliniporator Technical Sheet,” February 2018, http://igeamedical.com, http://www.igeamedical.com/sites/default/files/prodotti/synthesis-chemistry-and-physics-fight-cancer/files_allegati/cliniporatortechnicalsheet.pdf

[33] Schmidt G., Juhasz-Böss I., Solomayer E.-F., Herr D. (2014). Electrochemotherapy in breast cancer: a review of references. *Geburtshilfe und Frauenheilkunde*.

[34] Colombo G. L., Di Matteo S., Mir L. M. (2008). Cost-effectiveness analysis of electrochemotherapy with the Cliniporator™ vs. other methods for the control and treatment of cutaneous and subcutaneous tumors. *Therapeutics and Clinical Risk Management*.

[35] Campana L. G., Marconato R., Valpione S. (2017). Basal cell carcinoma: 10-year experience with electrochemotherapy. *Journal of Translational Medicine*.

[36] Brace C. L. (2009). Radiofrequency and microwave ablation of the liver, lung, kidney, and bone: what are the differences?. *Current Problems in Diagnostic Radiology*.

[37] Poulou L. S., Botsa E., Thanou I., Ziakas P. D., Thanos L. (2015). Percutaneous microwave ablation vs. radiofrequency ablation in the treatment of hepatocellular carcinoma. *World Journal of Hepatology*.

[38] Jahangeer S., Forde P., Soden D., Hinchion J. (2013). Review of current thermal ablation treatment for lung cancer and the potential of electrochemotherapy as a means for treatment of lung tumours. *Cancer Treatment Reviews*.

[39] Mavrogenis A. F., Angelini A., Vottis C. (2016). Modern palliative treatments for metastatic bone disease: awareness of advantages, disadvantages, and guidance. *The Clinical Journal of Pain*.

